# Other-Oriented Perfectionism in Children and Adolescents: Development and Validation of the Other-Oriented Perfectionism Subscale-Junior Form (OOPjr)

**DOI:** 10.1177/07342829211062009

**Published:** 2022-03-05

**Authors:** Paul L. Hewitt, Martin M. Smith, Gordon L. Flett, Ariel Ko, Connor Kerns, Susan Birch, Hira Peracha

**Affiliations:** 1 8166 University of British Columbia, Vancouver, BC, Canada; 2 7991 York University, Toronto, ON, Canada

**Keywords:** OOPjr, other-oriented perfectionism, children, CAPS

## Abstract

Research on adults indicates other-oriented perfectionism (requiring perfection from others) is associated with various consequential outcomes independent of self-oriented perfectionism (requiring perfection of the self) and socially prescribed perfectionism (believing others require perfection of the self). However, historically, the most widely used and researched measure of trait perfectionism in children, the Child-Adolescent Perfectionism Scale (CAPS), has omitted other-oriented perfectionism. In the present study, we address this by reporting on the multisource development and validation of the first self-report measure of other-oriented perfectionism specifically intended for youths: the Other-Oriented Perfectionism Subscale-Junior Form (OOPjr). Children (*N* = 107; Mage = 11.5, SD = 1.7) completed the OOPjr, CAPS, and measures of perfectionistic self-presentation, narcissism, social disconnection, depressive symptoms, and parental psychological control. Parents provided ratings of children’s self-oriented, socially prescribed, and other-oriented perfectionism. Psychometric analyses indicated the OOPjr is a homogenous and internally reliable scale that, when factor analyzed alongside the CAPS, displays measurement invariance across gender and replicates the three-factor solution found in adults. Furthermore, parent ratings of other-oriented perfectionism showed unique positive relationships with OOPjr scores, but not CAPS scores. Likewise, other-oriented perfectionism had independent positive relationships with narcissistic superiority and achievement-oriented parental psychological control, after controlling for self-oriented and socially prescribed perfectionism. Overall, our findings provide preliminary support for the use of the OOPjr as a measure of other-oriented perfectionism in youths.

## Other-Oriented Perfection-Junior

Perfectionism is a widespread and severe problem among adolescents and children. Indeed, Stornæs et al. (2019) studied 832 community adolescents and found 36% had high perfectionism. Similarly, in a sample of 319 children, Melero et al. (2020) concluded that 27.6% had high perfectionism. Likewise, when Törnblom et al. (2013, p. 248) conducted interviews with parents of adolescent boys who died by suicide, they found that 68.1% believed their child’s “high demands and expectations”—hallmarks of perfectionism—were contributing factors. Besides suicide, a wealth of evidence also implicates perfectionism in an array of other childhood difficulties, including depression (e.g., Hewitt et al., 2002), peer problems (Melero et al., 2020), clinically diagnosed anxiety (Mitchell et al., 2013), and self-harm (O’Connor et al., 2010). Accordingly, the high prevalence of perfectionism coupled with its pernicious effects underscores the urgent need for prevention, intervention, and treatment efforts (Flett & Hewitt, 2013). Yet, the success of such efforts hinges on the accuracy of our conceptualization and measurement of perfectionism.

### Conceptualizing Perfectionism

Hewitt et al.’s (2017) Comprehensive Model of Perfectionistic Behavior (CMPB) conceptualizes perfectionism as a multifaceted and multilevel personality style that permeates most behavior and has intertwined trait, self-presentational, and cognitive components (Hewitt, 2020). The trait component (Hewitt & Flett, 1991) reflects the deeply engrained requirement of perfection and distinguishes both the source and target of perfectionistic expectations. As such, there are three trait dimensions: self-oriented perfectionism (requiring perfection of the self), socially prescribed perfectionism (belief that others require perfection of the self), and other-oriented perfectionism (requiring perfection of other people). Whereas self-oriented and socially prescribed perfectionism involves a hostile internal dialogue regarding the need of the self and the needs of others for perfection, other-oriented perfectionism is particularly unique in that this need for perfection and hostility is directed outward onto other people. Next, whilst the trait components of the CMPB reflect what people have in terms of perfectionism, the self-presentational component reflects how people express perfectionism interpersonally. Thus, as with the trait component, the self-presentation component has three facets: perfectionistic self-promotion (i.e., promoting and presenting one’s perfection), non-display of imperfection (i.e., concealing behavioral displays of imperfection), and non-disclosure of imperfection (i.e., avoiding disclosing imperfections verbally). Finally, perfectionistic cognitions are seen by the CMPB as an internal expression of the need to be, or appear to be, perfect that involves self-directed dialogue, thoughts, and images^1^. Parenthetically, though all levels of the CMPB are important (see Hewitt et al., 2017), the present paper focuses on trait perfectionism.

### The Perfectionism Social Disconnection Model (PSDM)

The PSDM provides an in-depth, theory-driven account of perfectionism, its development, and its consequences through the lens of dynamic-relational theory (Hewitt et al., 2017). Though the PSDM has several hypotheses, it broadly theorizes that perfectionism makes it difficult for people to participate in and benefit from close relationships, which, in turn, engenders various forms of psychopathology. In support, self-oriented and socially prescribed perfectionism indirectly predicted longitudinal increases in various forms of psychopathology such as depressive symptoms (see Hewitt et al., 2017 for review). However, unlike self-oriented and socially prescribed perfectionism, other-oriented perfectionism is an inconsistent predictor of internalizing problems, perhaps due to the tendency for people higher in other-oriented perfectionism to externalize blame (Chen et al., 2017). As such, other-oriented perfectionism is absent from most tests of the PSDM.

Nonetheless, Sherry et al. (2016) maintain the tendency for researchers to overlook other-oriented perfectionism when studying the PSDM is ill-advised because “the recipients of perfectionistic demands may suffer more than the source of the perfectionistic demands” (p. 230). More specifically, they posited that like self-oriented and socially prescribed perfectionism, other-oriented perfectionism is associated with interpersonal dysfunction. But that, unlike self-oriented and socially prescribed perfectionism, the dysfunction generated by other-oriented perfectionism adversely impacts other people. In support, Hewitt et al. (1992) reported a link between other-oriented perfectionism and antisocial traits. Similarly, Stoeber (2014) found that, after controlling overlap among trait perfectionism dimensions, other-oriented perfectionism had unique positive relationships with Machiavellianism and psychopathy. Likewise, Smith and colleagues (2016) presented meta-analytic evidence that other-oriented perfectionism displays a unique positive relationship with narcissistic grandiosity independent of self-oriented and socially prescribed perfectionism. And Hewitt et al. (2021) found that patients with elevated other-oriented perfectionism received less positive clinician ratings as a consequence of clinician-rated hostility. Moreover, in direct support of Sherry et al. (2016), Smith et al. (2017) studied mother–daughter dyads and reported that the university-aged daughters of mothers higher in other-oriented perfectionism tended to experience longitudinal decreases in social self-esteem and, in turn, longitudinal increases in depressive symptoms. Considering these findings together, though all trait perfectionism dimensions are associated with interpersonal dysfunction, the dysfunction associated with other-oriented perfectionism is “dark” (Flett, Hewitt, & Sherry, 2016; Marcus & Zeigler-Hill, 2015).

### Trait Perfectionism in Children

As with adults, self-oriented and socially prescribed perfectionism are associated with maladjustment in children. Indeed, self-oriented and socially prescribed perfectionism correlate positively with depressive disorders in pre-adolescent boys and girls (Hewitt et al., 2002). Likewise, in children, depressive symptoms predict longitudinal increases in socially prescribed perfectionism 3 years later (Asseraf & Vaillancourt, 2015). Furthermore, socially prescribed perfectionism predicts internalizing problems in children as young as nine and continues to hinder adjustment up to 2 years later (Hong et al., 2017). Likewise, there is correlational and experimental evidence that self-oriented and socially prescribed perfectionism are tied to increased interpersonal difficulty, rejection sensitivity, social disconnection, eating disorder symptomology, depressive symptoms, and anxiety in young children (Magson et al., 2019).

However, perfectionism not only leaves children vulnerable to psychopathology, but also limits the success of psychotherapy. Indeed, in line with the adult literature (Hewitt et al., 2020), perfectionism-related attitudes predict non-responsiveness to treatments for adolescent depression (Jacobs et al., 2009). There is also theory and evidence suggesting that perfectionism co-occurs with overly controlling parenting behaviors (Flett et al., 2002; Hewitt et al., 2017). For instance, Kenney-Benson and Pomerantz (2005) observed mother–child dyads completing a homework task and found controlling maternal behavior correlated with children’s self-oriented and socially prescribed perfectionism. Curran (2018) found that parental psychological control displayed small-to-large positive relationships with self-oriented and socially prescribed perfectionism in adolescents. And Ko (2019) studied a gender-balanced sample of children and found that self-oriented and socially prescribed perfectionism had moderate, positive relationships with parental psychological control independent of parenting styles. Accordingly, theory and evidence suggest children higher in self-oriented and socially prescribed perfectionism have overly controlling parents. However, the extent to which these findings apply to other-oriented perfectionism is unclear.

### Advancing Research on Trait Perfectionism in Children

Researchers studying perfectionism in youths initially administered adult measures to child and adolescent samples (e.g., Hankin et al., 1997; Parker, 2002). However, adult measures are not appropriate for youths due to a lack of validation, item appropriateness, and developmental differences in the constructs measured (National Council on Measurement in Education, 1999). Thus, Flett et al. (2000) addressed this by developing the Child-Adolescent Perfectionism Scale (CAPS; Flett, Hewitt, Besser, et al., 2016). The CAPS is the youth version of the Multidimensional Perfectionism Scale (MPS) and derives from Hewitt and Flett’s (1991) conceptualization of trait perfectionism. Consequently, both the MPS and CAPS assess self-oriented and socially prescribed perfectionism. Yet, unlike its adult counterpart, other-oriented perfectionism is absent from the CAPS. The reason being that, in Flett et al.’s (2000) words, “at present, the CAPS does not include a subscale measuring other-oriented perfectionism because our examination of the developmental literature revealed…few references to other-oriented perfectionism.” Now, though this decision was defensible, it is also no longer tenable.

First, in older adolescents, other-oriented perfectionism has a unique positive relationship with parental psychological control, independent of socially prescribed perfectionism (Curran et al., 2017). Similarly, other researchers have demonstrated that in older adolescents other-oriented perfectionism can be measured reliably (e.g., Mallinson & Hill, 2011; Randall et al., 2015). That said, a limitation of these studies is that they used an adult measure of other-oriented perfectionism because an age-appropriate measure had not been developed. Second, there is indirect evidence suggesting other-oriented perfectionism is present in young children. Namely, research suggests narcissism can be reliably assessed in children as young as seven (Barry et al., 2003; Brummelman et al., 2015; Thomaes et al., 2008). Narcissism overlaps conceptually and empirically with other-oriented perfectionism (Smith, Sherry, Chen, et al., 2016). Moreover, the measures used to assess narcissism in children contain item content with clear parallels to other-oriented perfectionism. For instance, consider the Narcissistic Personality Inventory for Children (Barry et al., 2003) items, “I want to control other people” and “I expect to get a lot from other people.” Third, Hewitt et al. (2017) provided an in-depth theoretical account of how other-oriented perfectionism can emerge in childhood. Briefly, they theorized that other-oriented perfectionism emerges in early childhood due to an asynchrony characterized by caregivers who are incapable or unwilling to meet their child’s needs. These unmet needs, in turn, “lay the foundation for interpersonal distance, a constricted capacity for empathy, and a determination to control the child’s relational world by insisting that his or her expectations are met in a highly specific manner” (Hewitt et al., 2017; p. 122–123). Nonetheless, at present, we lack an age-appropriate means of studying the development of other-oriented perfectionism.

### The Present Study

Against this background, we aimed to catalyze research on the expression and consequences of other-oriented perfectionism in children by developing and validating the first measure of other-oriented perfectionism specifically intended for youths—the Other-Oriented Perfectionism Subscale-Junior Form (OOPjr). Besides evaluating psychometrics, we will assess validity in several ways. Hewitt et al.’s (2017) CMPB conceptualizes perfectionism as having overlapping trait and self-presentational components. As such, we anticipate other-oriented perfectionism will show significant positive associations with self-oriented perfectionism, socially prescribed perfectionism, and the three perfectionistic self-presentation facets (Hewitt et al., 2017). Likewise, we expect other-oriented perfectionism will have a unique positive relationship with parent ratings of other-oriented perfectionism beyond parent ratings of self-oriented and socially prescribed perfectionism. In other words, we expect parents will not only be aware of their child’s other-oriented perfectionism but will be able to distinguish it from their self-oriented and socially prescribed perfectionism. In consideration of theory (Flett et al., 2002) and evidence (e.g., Curran, 2018; Smith et al., 2017), we also expect that other-oriented perfectionism will explain incremental variance in parental psychological control beyond self-oriented and socially prescribed perfectionism. Furthermore, based on the adult literature (e.g., Chen et al., 2017), we anticipate that, unlike self-oriented and socially prescribed perfectionism, the relationship between other-oriented perfectionism and depressive symptoms will be negligible. As well, in consideration of theory (Hewitt et al., 2017) and evidence (Smith, Sherry, Chen, et al., 2016), we expect that other-oriented perfectionism will display unique positive relationships with narcissistic superiority and exploitativeness, independent of self-oriented and socially prescribed perfectionism. Also, as noted by Sass (2011), demonstrating that a scale performs equally across groups is a key component of validity. Though research testing gender differences in perfectionism in child-adolescent samples is scarce, Hewitt et al. (2002) found that boys tended to score higher on socially prescribed perfectionism than girls. Accordingly, consistent with best practice guidelines for scale development (Tock & Moxley, 2018), we examined the extent to which the OOPjr displays measurement invariance across gender. Lastly, we anticipated that factor analyzing the OOPjr alongside the CAPS would reveal that the same three factors found in adults (i.e., other-oriented perfectionism, self-oriented perfectionism, and socially prescribed perfectionism) would emerge in our child-adolescent sample. If evidence in support of this contention is found, it would suggest that researchers and clinicians can assess the entirety of trait perfectionism in children by administering the OOPjr alongside the CAPS.

## Methods

### Participants

A sample of 107 parent–child dyads was recruited through the University of British Columbia’s Early Development Research Group (EDRG). The EDRG shares a database containing information on families who previously consented to be contacted for research studies. Any child or adolescent between the ages of 8–15 with a primary caregiver from [Masked for Review] was contacted. Children averaged 11.5 years of age (*SD* = 1.7) and 61.5% were female. Most children (54.2%) were Caucasian, with the remainder identifying as Mixed Race (18.7%), Chinese (10.3%), South Asian (6.5%), Southeast Asian (2.8%), Middle Eastern (1.9%), or “other” (4.7%). The majority of children (95%) spoke English as their first language, and the remainder reported having spoken English for over 5 years. Parents averaged 46.2 years of age (*SD* = 5.3, range = 32–64), and most were the child’s biological mother (86%). Overall, 4.7% of parents had an average household income of less than $25,000, 7.5% had an income between $25,001 and $50,000, 27.1% had an income of $50,001 to $100,00, and 60.7% had an income of more than $100,001. The majority of parents (98.1%) reported living in Canada for more than 10 years, with the remainder living in Canada for more than 5 years.

### Measures

#### Initial Item Pool for the OOPjr

Other-oriented perfectionism was operationalized as requiring perfection from other people (Hewitt & Flett, 1991; see Supplemental Material for a more detailed operational definition). Our aim was to develop a set of homogenous items derived from theory, evidence, and clinical observations that assess other-oriented perfectionism in child-adolescent samples via one factor (Clark & Watson, 1995; Reise et al., 2000). To this end, consistent with Jackson’s (1970) recommendations for scale development, the first author created an initial pool of 30-items through a comprehensive literature review and item creation by trained item-writers based on this review. Next, the first and second authors removed items judged as redundant, developmentally inappropriate, ambiguous, or problematic (e.g., double-barreled), leaving a total of 17-items. We then administered these 17-items to children who rated each item using a 5-point scale from 1 (*not at all*) to 5 (*extremely*). As indicated below, the final OOPjr consisted of 10-items and had good internal consistency (α = .90).

#### Trait Perfectionism

Self-oriented perfectionism and socially prescribed perfectionism were measured using the CAPS (Flett et al., 2000; Flett, Hewitt, Besser, et al., 2016). The CAPS is a measure of self-oriented perfectionism (12-items; e.g., “When I do something, it has to be perfect”) and socially prescribed perfectionism (10-items; e.g., “My family expects me to be perfect”). Children responded to CAPS items using a 5-point scale from 1 (*False–Not at all like me*) to 5 (*Very true of me*). The CAPS is the most widely used and validated measure of perfectionism in youths. Asseraf and Vaillancourt (2015) studied children in Grade 7 and found one-year test–retest reliabilities of .66 and .60, for self-oriented and socially prescribed perfectionism, respectively. Magson et al. (2019) reported that, in a sample of 11-year-old children, self-oriented and socially prescribed perfectionism had Cronbach’s alphas of .80 and .88, respectively. In the present study, self-oriented and socially prescribed perfectionism had Cronbach’s alphas of .90 and .86.

#### Perfectionistic Self-Presentation

Perfectionistic self-presentation was measured using the Perfectionistic Self-Presentation Scale–Junior Form (PSPSjr; Hewitt et al., 2011). The PSPSjr is a measure of perfectionistic self-promotion (8-items; e.g., “If I seem perfect, other people will like me more”), non-display of imperfection (6-items; e.g., “I do not want my friends to see even one of my bad points), and non-disclosure of imperfection (4-items; e.g., “I should always keep my problems secret”). Children responded to the PSPjr using a 5-point scale from 1 (*Disagree Strongly*) to 5 (*Agree Strongly*). Hewitt et al. (2011) reported coefficient alphas of .92, .82, and .72 for perfectionistic self-promotion, non-display of imperfection, and non-disclosure of imperfection in a heterogeneous sample of youths aged 8 to 17. In the present study, the internal consistency of perfectionistic self-promotion, non-display of imperfection, and non-disclosure of imperfection were .90, .74, and .66.

#### Narcissism

Narcissism was measured using Ang and Raine’s (2009) Narcissistic Personality Questionnaire for Children-Revised (NPQC-R). The NPQC-R is a measure of superiority (6-items, e.g., “I always know what I am doing”) and exploitativeness (6-items; e.g., “I can make people believe anything I want them to”). Children responded to NPQC-R using a 5-point scale from 1 (*not at all like me*) to 5 (*completely like me*). Ang and Raine (2009) found two-week test–retest reliabilities of .67–.85 across two independent samples of adolescents. In the present study, narcissistic superiority and narcissistic exploitativeness had a Cronbach’s alpha of .80 and .64.

#### Social Disconnection

Social disconnection was measured using Lee et al.’s (2001) Social Connection Scale-Revised (SCS-R; 20-items). The SCS-R assesses the degree to which youth feel connected to others. Children responded to SCS-R items (e.g., “I don’t feel that I participate with anyone or any group”) using a six-point scale from 1 (*strongly agree*) to 6 (*strongly disagree*). Chen et al. (2012) reported the SCS-R had a Cronbach’s alpha of .70 in a sample of adolescents. In the present study, the SCS-R had a Cronbach’s alpha of .87. Items were reversed such that higher scores indicate greater social disconnection.

#### Depression

Depressive symptoms were assessed using the Kroenke et al. (2001) Physical Health Questionnaire depression subscale (PHQ-9). The PHQ-9 assesses each of the 9 DSM-IV criteria for depression. Children responded to PHQ-9 items (e.g., “Feeling down, depressed, hopeless”) using a 4-point scale from 0 (*not at all*) to 3 (*nearly every day*). Evidence supporting the reliability and validity of the PHQ-9 as a measure of depression severity in children are presented in detail in Johnson et al. (2002).

#### Psychological Control

Dependency-oriented and achievement-oriented parental psychological control were measured using Soenens et al.’s (2010) Dependency-Oriented and Achievement-Oriented Psychological Control Scale (DAPCS). The DAPCS is comprised of a dependency-oriented achievement subscale (10-items; e.g., “My parents are only happy with me if I rely exclusively on them for advice”) and an achievement-oriented subscale (5-items; e.g., “My parents are only friendly with me if I excel in everything I do”). Children responded to DAPCS items using a 5-point scale from 1 (*not at all*) to 5 (*always*). Soenens et al. (2010) reported Cronbach’s alphas between .76 and .92 in adolescents. In the current study, Cronbach’s alphas for the dependency-oriented and achievement-oriented subscales were .86 and .85.

#### Parent ratings

Parents were given definitions of self-oriented, other-oriented, and socially prescribed perfectionism (see Supplemental Material) and were asked to rate their child on these dimensions by responding to single items using a 4-point rating scale from 0 (*Not at all*) to 3 (*Extremely*).

## Results

### Item Selection, Reduction, Factor Analysis, and Measurement Invariance

The Kaiser–Meyer–Olkin measure of sampling adequacy was .89 indicating the data were suitable for factor analysis. Exploratory factor analysis of the pool of 17 items indicated a one factor solution had acceptable fit: WLSMV χ^2^(119) = 195.47, RMSEA = .079 (90% CI = .059; .099), CFI = .948, and TLI = .941. Even so, the addition of a second factor improved fit significantly: WLSMV Δχ^2^(16) = 57.69, *p* < .001. Conversely, the addition of a third factor did not improve fit significantly: Δχ^2^(15) = 25.042, *p* = .05. Eigenvalues for the 17-item solution were 9.3, 1.9, 1.1, and .9. Inspection of the content of the items with the highest loading on the second factor suggests that the second factor was capturing content that was not per se perfectionistic (e.g., “I expect other people to do their absolute best,” “If I am trying to be perfect, others should also try to be perfect,” “The kids in my class should try harder to be perfect”). Accordingly, to increase homogeneity and remove irrelevant content, we specified a two-factor solution and removed seven items with loadings below .40 on the first factor or loadings above .40 on the second factor^2^. This resulted in the final 10-item OOPjr. A second exploratory factor analysis was then conducted to evaluate the unidimensionality of the OOPjr. The fit of one-factor solution was acceptable: WLSMV χ^2^(35) = 44.64, RMSEA = .052 (90% CI = .000; .093), CFI = .990, and TLI = .987, and the inclusion of a second factor did not result in a significant improvement in fit: WLSMV Δχ^2^(9) = 15.02, *p* = .095. Moreover, the first factor had an eigenvalue of 6.70, whereas the second factor had an eigenvalue of .90, again suggesting a one-factor solution. Loadings and items are shown in Table 1.

**Table 1.** CAPS-OOP Items and Factor Loadings From the Exploratory Factor Analysis.

	Factor 1
Other-oriented perfectionism	
1. I do not like to be friends with anyone who is not perfect	.95
2. If other kids aren’t perfect, I don’t like them	.92
3. People who want to be my friend need to be perfect	.87
4. It is important that people I am close to are perfect	.85
5. I need my family members to be perfect	.84
6. Everything that others do must be perfect	.79
7. I get upset when other kids aren’t perfect	.76
8. I need my friends to be perfect	.74
9. I think less of my classmates if they make mistakes	.69
10. I expect my friends to be the best, not second best	.67

*Note.* All loadings are significant at *p* < .001.

Subsequently, we combined the OOPjr with the CAPS and used confirmatory factor analysis (CFA) with weighted least squares estimation (WLSMV) to evaluate the fit of the three-factor solution found in adults (see Supplemental Figure 1). Model fit was acceptable: WLSMV χ^2^(461) = 598.41, RMSEA = .053 (90% CI = .040; .065), CFI = .952, and TLI = .948. As such, we proceeded to test measurement invariance across gender. Constraining factor loadings to be equal across boys and girls did not lead to a significant loss of fit (MLR Δχ2 [28] = 38.81, *p* = .084)^3^. Similarly, constraining item thresholds and factor loadings to be equal across boys and girls did not result in a significant loss of fit (MLR Δχ^2^ [56] = 71.71, *p* = .077). Accordingly, this suggests that the three-factor solution obtained for the OOPjr and CAPS replicates across male and female children. Moreover, it suggests that researchers who wish to assess other-oriented, self-oriented, and socially prescribed perfectionism in children can do so reliably by administering OOPjr items alongside CAPS items.

Consistent with Harrington and Follett (1984), we evaluated the extent to which children can read and understand OOPjr items by calculating two readability scores (see Klare, 1984): the Flesh-Reading Ease (FRE = .87; Klare, 1974) and Flesh Grade Level (FGL = 3.5; Klare, 1975). This suggests the OOPjr items can be understood by the average child in grades four and above.

### Descriptive Statistics and Preliminary Analysis

Less than 5% of data points were missing. We used full information maximum likelihood estimation to handle missing data (Little et al., 2014). Alpha reliabilities, bivariate correlations, and descriptive statistics are in Table 2.

**Table 2.** Bivariate Correlations, Means, Standard Deviations, and Cronbach’s alpha.

	1	2	3	4	5	6	7	8	9	10	11	12	13	14	
1. Other-oriented perfectionism	—	—	—	—	—	—	—	—	—	—	—	—	—	—	—
2. Self-oriented perfectionism	.26^**^	—	—	—	—	—	—	—	—	—	—	—	—	—	—
3. Socially prescribed perfectionism	.45^***^	.63^***^	—	—	—	—	—	—	—	—	—	—	—	—	—
4. Perfectionistic self-presentation	.45^***^	.61^***^	.59^***^	—	—	—	—	—	—	—	—	—	—	—	—
5. Non-display of imperfection	.24^*^	.64^***^	.47^***^	.69^***^	—	—	—	—	—	—	—	—	—	—	—
6. Non-disclosure of imperfection	.21^*^	.43^***^	.39^***^	.55^***^	.57^***^	—	—	—	—	—	—	—	—	—	—
7. Narcissistic superiority	.20^*^	−.03	.05	−.13	−.20^*^	–.21^*^	—	—	—	—	—	—	—	—	—
8. Narcissistic exploitativeness	.07	.34^***^	.33^***^	.17	.12	.22^*^	.31^**^	—	—	—	—	—	—	—	—
9. Psychological control	.41^***^	.39^***^	.44^***^	.40^***^	.41^***^	.36	−.03	.08	—	—	—	—	—	—	—
10. Dependency-oriented control	.34^**^	.34^***^	.40^***^	.36^***^	.39^***^	.34^***^	−.06	.04	.98^***^	—	—	—	—	—	—
11. Achievement-oriented control	.43^***^	.39^***^	.41^***^	.39^***^	.31^**^	.37^***^	−.01	.08	.76^***^	.61^***^	—	—	—	—	—
12. Depressive symptoms	.08	.39^***^	.34^***^	.32^**^	.49^***^	.44^***^	−.32^**^	.08	.43^***^	.44^***^	.31^**^	—	—	—	—
13. Social disconnection	.27^**^	.31^**^	.32^**^	.44^***^	.48^***^	.46^***^	−.44^***^	−.10	.34^***^	.36^***^	.22^*^	−.51^***^	—	—	—
14. Age	−.24^*^	.38^***^	.22^*^	.20^*^	.22^*^	.12	−.07	.29^**^	−.09	−.12	−.05	.08	−.02	—	—
15. Gender	.06	−.14	.08	−.07	−.14	−.01	.06	.01	.14	.11	.09	−.07	.16	−.07	—
*M*	13.1	39.6	23.4	18.7	18.8	10.6	18.7	13.4	22.7	17.7	5.1	5.2	84.8	11.5	1.4
*SD*	5.5	8.2	7.9	7.1	4.6	3.1	4.6	4.0	0.5	6.3	1.8	5.4	15.6	1.7	0.5
Min	10	25	10	8	10	4	7	6	15	11	4	0	39	8.0	1
Max	50	56	43	39	29	18	30	23	50	42	13	25	119	15.0	2
Cronbach’s alpha (α)	.86	.90	.86	.90	.74	.66	.80	.64	.86	.85	.70	.88	.87	—	—

*Note.* Pairwise deletion (*N* = 99–107).

^*^p < .05; ^**^p < .01; ^***^p <.001.

Consistent with research on other-oriented perfectionism in adults (see Hewitt et al., 2017 for review), other-oriented perfectionism displayed small-to-large positive correlations with self-oriented perfectionism, socially prescribed perfectionism, and perfectionistic self-presentation facets (*r* = .20 to .45). Furthermore, consistent with the PSDM, other-oriented perfectionism had a small positive relationship with social disconnection (*r* = .27). Likewise, consistent with the adult literature (Smith, Sherry, Rnic, et al., 2016; Smith et al., 2021), self-oriented and socially prescribed perfectionism, but not other-oriented perfectionism, had moderate positive relationships with depressive symptoms (*r* = .34 to .39). The OOPjr also had a Flesh-Reading Ease score of 86.7 and an FGL (Klare, 1975) score of 3.5. This indicates the OOPjr is written in plain language that can be understood by the average child in grades 4 and above.

### Construct Validity

**Figure 1.**
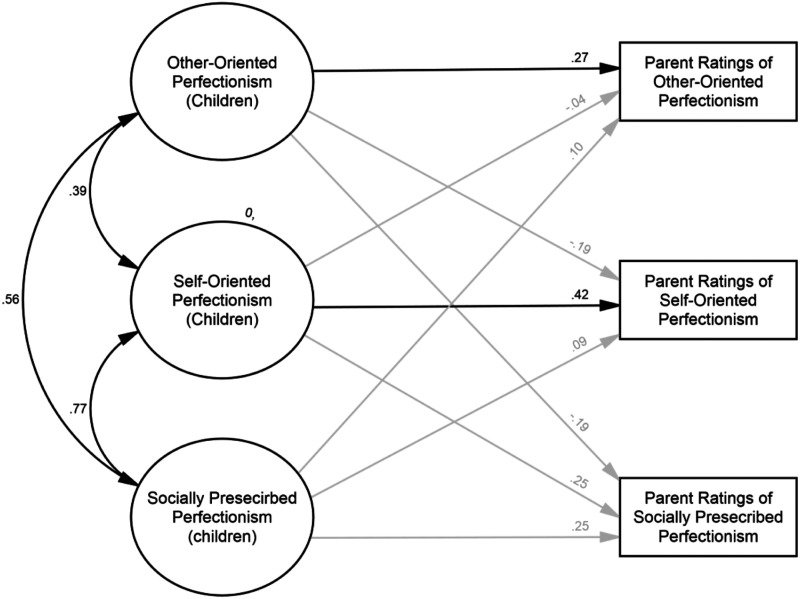
Structural model. Ovals represent latent variables. Rectangles represent observed variables. Estimates are standardized. Error terms and factor loadings are not displayed for clarity. The double-headed black arrows indicate a significant correlation (*p* < .05). Single-headed black arrows represent significant paths (*p* < .05). Single-headed grey arrows represent non-significant paths (*p* > .05).

We used structural equation modeling to evaluate parent–child agreement (see Figure 1). A multitrait multimethod matrix is included in our supplemental material. Model fit was acceptable: WLSMV χ^2^(548) = 686.59, RMSEA = .049 (90% CI = .036; .060), CFI = .951, and TLI = .947. As anticipated, other-oriented perfectionism had a significant positive relationship with parent ratings of other-oriented perfectionism (*β* = .27, *p* = .022), but not parent ratings of self-oriented (*β* = −.19, *p* = .135) or socially prescribed perfectionism (*β* = −.04, *p* = .796). Similarly, self-oriented perfectionism had a significant positive relationship with parent ratings of self-oriented perfectionism (*β* = .42, *p* = .005), but not socially prescribed perfectionism (*β* = .25, *p* = .090) or other-oriented perfectionism (*β* = −.04, *p* = .796). In contrast, socially prescribed perfectionism was not significantly related to parent ratings of socially prescribed perfectionism (*β* = .09, *p* = .573), other-oriented perfectionism (*β* = .10, *p* = .652), or self-oriented perfectionism (*β* = .09, *p* = .640).

### Incremental Validity

**Figure 2.**
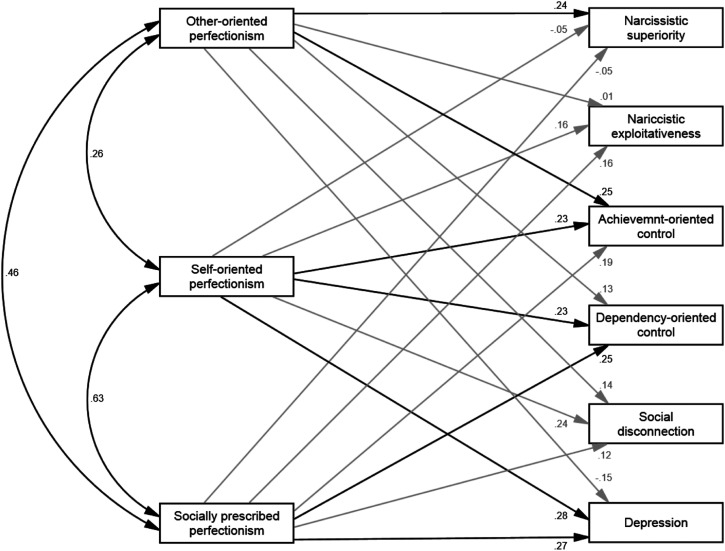
Path diagram depicting association among variables. Estimates are standardized. Rectangles represent observed variables. Age, error terms, and correlations among error terms omitted for clarity. The path from age to narcissistic superiority was β = .02 [95% CI: −.21, .24]. The path from child age to narcissistic exploitativeness was β = .20 [95% CI: .00, .41]. The path from age to achievement-oriented control was β = −.15 [95% CI: −.34, .05]. The path from age to dependency-oriented control was β = −.22 [95% CI: −.42, −.02]. The path from age to social disconnection was β = −.07 [95% CI: −.28, .14]. The path from age to depressive symptoms was β = −.13 [95% CI: −.34, .08]. The double-headed black arrows indicate a significant correlation (*p* < .05). Single-headed black arrows represent significant paths (*p* < .05). Single-headed grey arrows represent non-significant paths (*p* > .05).

We conducted path analysis to examine in a single model the extent to which other-oriented perfectionism incrementally adds to the predictions of dependency-oriented psychological control, achievement-oriented psychological control, narcissistic superiority, narcissistic exploitativeness, social disconnection, depression beyond self-oriented perfectionism, socially prescribed perfectionism, and age (see Figure 2). Given the significant small negative relationship between age and other-oriented perfectionism and the small-to-moderate positive relationships between age, self-oriented perfectionism, and socially prescribed perfectionism, age was included as a covariate^4^. Model fit was just-identified (*df* = 0).

After removal of variance attributable to self-oriented and socially prescribed perfectionism, as well as age, other-oriented perfectionism had small positive relationships with achievement-oriented psychological control (*β* = .24, *p* = .018) and narcissistic superiority (*β* = .24, *p* = .018). In contrast, though other-oriented perfectionism displayed small significant relationships with dependency-oriented control and social disconnection (Table 1), these relationships ceased to be significant after accounting for variance attributable to self-oriented, socially prescribed perfectionism, and age (Figure 2).

## Discussion

In the present study, we reported on the development and validation of a measure of other-oriented perfectionism intended for use in children. Based on a sample of children of approximately 11 years of age, results suggest the OOPjr is homogenous, unidimensional, and internally consistent (Comrey, 1988). The three trait perfectionism factors found in adults—other-oriented perfectionism, self-oriented perfectionism, and socially prescribed perfectionism—explained most of the common variance among OOPjr and CAPS items. Thus, Hewitt and Flett’s (1991) conceptualization of trait perfectionism as involving three dimensions appears to generalize to children. Results also support the validity of the OOPjr as a measure of other-oriented perfectionism and our contention that in children, other-oriented perfectionism is associated with, but distinct from, self-oriented and socially prescribed perfectionism.

### An Improved Understanding of Other-Oriented Perfectionism in Children

We found several pieces of evidence supporting the validity of the OOPjr. Perhaps the most compelling being the convergence between other-oriented perfectionism and parent ratings of other-oriented perfectionism (see Figure 1). This result suggests that children express other-oriented perfectionism in ways that involve behaviors observable to parents. Moreover, it implies parents can distinguish their child’s other-oriented perfectionism from their self-oriented and socially prescribed perfectionism. Parenthetically, a similar pattern was observed for self-oriented, but not socially prescribed, perfectionism (see Figure 1). This may reflect other-oriented and self-oriented perfectionism involving behaviors that are more observable to parents (Klonsky et al., 2002).

Additionally, in line with Curran et al. (2017), findings revealed that other-oriented perfectionism was a robust predictor of parental psychological control even after accounting for overlap among trait perfectionism dimensions. This is consistent with longstanding theoretical accounts on the development of perfectionism. For instance, Horney et al. (1939) observed that perfectionism develops in children with “self-righteous parents who exert unquestionable authoritative sway,” (p. 218) and Hamachek (1978) theorized perfectionism develops in response to parental behaviors characterized by “inconsistent approval” (p. 388). However, our results add greater specificity to these accounts by showing that different forms of perfectionism are differentially related to subtypes of psychological control. Namely, after accounting for overlap among trait perfectionism dimensions, other-oriented perfectionism appears most relevant to achievement-oriented psychological control, whereas socially prescribed perfectionism appears most germane to dependency-oriented psychological control.

Moreover, other-oriented perfectionism was uniquely associated with narcissistic superiority beyond self-oriented and socially prescribed perfectionism. This suggests that as with adults (Smith, Sherry, Chen, et al., 2016), children higher in other-oriented perfectionism believe they deserve special treatment and show less concern and empathy for other people (Thomaes et al., 2008). In contrast, unexpectedly, other-oriented perfectionism was not associated with narcissistic exploitativeness. This might reflect other-oriented perfectionism in adulthood being expressed differently than in childhood. Alternatively, the low internal consistency of the narcissistic exploitativeness subscale used (i.e., .64) may have prevented us from detecting this relationship.

Consistent with the PSDM (Hewitt et al., 2017), all trait perfectionism dimensions displayed small-to-moderate positive bivariate relationships with social disconnection. However, other-oriented perfectionism ceased to be a significant predictor of social disconnection after variance attributable to self-oriented and socially prescribed perfectionism was taken into account. Similarly, unlike self-oriented and socially prescribed perfectionism, other-oriented perfectionism had a negligible relationship with depressive symptoms. Thus, as with adults (Hewitt & Flett, 1993), by externalizing blame and distress, other-oriented perfectionism may buffer against depressive symptoms in children (Chen et al., 2017).

Regardless, consistent with the notion that measures developed for and used with adults are inappropriate for children because of developmental differences, reading ability, and comprehension (Eiser & Morse, 2001), the OOPjr represents a specific tool expressly intended to assess other-oriented perfectionism in children. This, in turn, allows for the investigation of several intriguing and vital questions. For example, evidence from research in adults is accumulating that the recipients of perfectionistic demands often suffer more than the source (e.g., Hewitt et al., 1995; Smith et al., 2017, 2019). Thus, research testing the extent to which one child’s other-oriented perfectionism impacts another child’s mental health would incrementally advance our understanding of the interpersonal consequences of perfectionism in youths. Alternatively, the adult literature suggests other-oriented perfectionism is associated with hostile and calculating behaviors, interpersonal dysfunction, and the use of aggressive humor (e.g., Hewitt, Smith, et al., 2020; Stoeber, 2014; Stoeber et al., 2021). And the developmental literature has also indicated that both the perpetrators and victims of bullying are at increased risk for mood disorders (Takizawa et al., 2014) and suicide (Cha et al., 2018). Moreover, research suggests that socially prescribed perfectionism predicts peer victimization in adolescents (Farrell & Vaillancourt, 2019). Thus, we maintain that it is highly likely that, as with adults, the link reported between socially prescribed perfectionism and peer victimization in children has less to do with socially prescribed perfectionism and more to do with socially prescribed perfectionism’s overlap with other-oriented perfectionism (Stoeber, 2014). Lastly, other-oriented perfectionism confers risk for a poor treatment outcome in adults (Hewitt, Smith, et al., 2020). As such, studying a clinical sample of youths and testing the extent to which other-oriented perfectionism limits the success of psychotherapy could prove important. Studying OOP in youths is also beneficial because of the possibility that intervening earlier in development might prove more fruitful than adult treatment approaches’ or something along the lines that children may be more malleable or intervening earlier before these patterns become especially established

### Limitations and Future Directions

The results of the present study should be considered in light of its limitations. First, the extent to which its factor structure holds across independent groups is unclear. Additionally, though our sample size is consistent with simulation research suggesting a 10:1 ratio of new items to participants (Costello & Osborne, 2005), other simulation research suggests correlations stabilize when samples greater than 250 are examined (Schönbrodt & Perugini, 2013). Furthermore, though we found evidence that the OOPjr displays measurement invariance across gender (Reise et al., 2000), we lacked the sample size needed to test measurement invariance across young children and older adolescents and across children of different cultural backgrounds. Thus, future research might consider investigating the extent to which the OOPjr replicates and displays measurement invariance across age, gender, and ethnicity using a large child-adolescent sample. Additionally, the mean for other-oriented perfectionism was lower than the mean for self-oriented and socially prescribed perfectionism. As such, relative to other trait perfectionism dimensions, other-oriented perfectionism may be less prominent in children. Nonetheless, it was still observable and uniquely associated with various outcomes.

## Concluding Remarks

In the present study, we reported on the development of a new measure specifically developed to assess other-oriented perfectionism in children—the OOPjr. Overall, results suggest other-oriented perfectionism is a distinct externalizing form of perfectionism that is not subsumed by self-oriented or socially prescribed perfectionism and displays theoretically relevant relationships with narcissistic superiority, achievement-oriented psychological control, and parent ratings. As such, the OOPjr appears to be a promising measure of individual differences in the outward expression of perfectionism that will allow developmental researchers, for the first time, to assess the totality of trait perfectionism when used alongside the CAPS. This will enable a more nuanced understanding of the development of perfectionism, which in turn may enhance our ability to detect, treat, and study this pernicious personality trait.

## Supplemental Material

sj-pdf-1-jpa-10.1177_07342829211062009 – Supplemental Material for Other-Oriented Perfectionism in Children and Adolescents: Development and Validation of the Other-Oriented Perfectionism Subscale-Junior Form (OOPjr) Click here for additional data file. Supplemental Material, sj-pdf-1-jpa-10.1177_07342829211062009 for Other-Oriented Perfectionism in Children and Adolescents: Development and Validation of the Other-Oriented Perfectionism Subscale-Junior Form (OOPjr) by Paul L. Hewitt, Martin M. Smith, Ariel Ko, Connor Kerns, Susan Birch, Hira Peracha and Gordon L. Flett in Journal of Psychoeducational Assessment

sj-pdf-2-jpa-10.1177_07342829211062009 – Supplemental Material for Other-Oriented Perfectionism in Children and Adolescents: Development and Validation of the Other-Oriented Perfectionism Subscale-Junior Form (OOPjr) Click here for additional data file. Supplemental Material, sj-pdf-2-jpa-10.1177_07342829211062009 for Other-Oriented Perfectionism in Children and Adolescents: Development and Validation of the Other-Oriented Perfectionism Subscale-Junior Form (OOPjr) by Paul L. Hewitt, Martin M. Smith, Ariel Ko, Connor Kerns, Susan Birch, Hira Peracha and Gordon L. Flett in Journal of Psychoeducational Assessment

sj-pdf-3-jpa-10.1177_07342829211062009 – Supplemental Material for Other-Oriented Perfectionism in Children and Adolescents: Development and Validation of the Other-Oriented Perfectionism Subscale-Junior Form (OOPjr) Click here for additional data file. Supplemental Material, sj-pdf-3-jpa-10.1177_07342829211062009 for Other-Oriented Perfectionism in Children and Adolescents: Development and Validation of the Other-Oriented Perfectionism Subscale-Junior Form (OOPjr) by Paul L. Hewitt, Martin M. Smith, Ariel Ko, Connor Kerns, Susan Birch, Hira Peracha and Gordon L. Flett in Journal of Psychoeducational Assessment

sj-pdf-4-jpa-10.1177_07342829211062009 – Supplemental Material for Other-Oriented Perfectionism in Children and Adolescents: Development and Validation of the Other-Oriented Perfectionism Subscale-Junior Form (OOPjr) Click here for additional data file. Supplemental Material, sj-pdf-4-jpa-10.1177_07342829211062009 for Other-Oriented Perfectionism in Children and Adolescents: Development and Validation of the Other-Oriented Perfectionism Subscale-Junior Form (OOPjr) by Paul L. Hewitt, Martin M. Smith, Ariel Ko, Connor Kerns, Susan Birch, Hira Peracha and Gordon L. Flett in Journal of Psychoeducational Assessment

## Notes

1. At present, a self-report measure of perfectionistic cognitions intended for youths has not been developed.

2. We present loadings for the two-factor solution in our supplemental material.

3. We were able to use WLMSV estimation as only four out of five of the possible response options for one OOPjr item were selected in boys.

4. The inclusion of age as a covariate resulted in no substantive differences in the magnitude or significance of findings.
