# Supplementary material for: Other-Oriented Perfectionism in Children and Adolescents: Development and Validation of the Other-Oriented Perfectionism Subscale-Junior Form (OOPjr)
Source: J Psychoeduc Assess. 2022 Mar 5;40(3):327–45. doi: 10.1177/07342829211062009 (PMC9092920; doi:10.1177/07342829211062009)
Supplement: sj-pdf-1-jpa-10.1177_07342829211062009 – Supplemental Material for Other-Oriented Perfectionism in Children and Adolescents: Development and Validation of the Other-Oriented Perfectionism Subscale-Junior Form (OOPjr) [file sj-pdf-1-jpa-10.1177_07342829211062009.pdf]

Supplemental Material:
Definitions of Perfectionism Dimensions Provided to Parents

Self-oriented Perfectionism: Self-oriented perfectionism involves perfectionistic behavior that relates to, or is directed toward the self. Individuals who have high levels of self-oriented perfectionism have perfectionistic or unrealistic self-expectations for themselves and place a great deal of importance on attaining perfection. They are preoccupied with their own demands for perfection and constantly require perfection from themselves in most aspects of their functioning. In addition, these people spend a great deal of time comparing their performance with internal standards, believing that they should never make mistakes. Because they hate to fail at anything, they tend to avoid things that can't be done well, and average performance or any performance less than perfect is unacceptable. Not surprisingly, these individuals engage in a lot of self-punishment and self-criticism and focus on negative aspects of their own performance to the exclusion of positive aspects. Finally, because high levels of success are what they always expect of themselves, in times of success, there is little self-reward or congratulations extended to the self.

Other-Oriented Perfectionism: Other-oriented perfectionism involves perfectionistic behavior, as described above, but the perfectionism is directed toward other people rather than to the self. Individuals who have high levels of other-oriented perfectionism have perfectionistic or unrealistic expectations for others and place a great deal of importance on others successfully attaining perfection. Not surprisingly, these individuals often engage in a lot of criticism and punishment of others and focus on negative aspects of others' performance to the exclusion of positive aspects. Finally, because high levels of success are what they always expect of others, in times of success, there is little reward administered or congratulations extended to the other person or persons.

Socially prescribed Perfectionism: Socially prescribed perfectionism involves perceiving that other people have perfectionistic expectations for one's own behavior and that other people expect or want the socially prescribed perfectionist to be perfect. Thus, self-worth is dependent on meeting other's expectations of perfection. In general, these people believe it is crucial to attain the perfection that they believe others expect. Because the socially prescribed perfectionist thinks others' acceptance of them depends on their own performance, making mistakes or appearing anything less than perfect is highly unacceptable. When these individuals do attain standards or perform well on some task, they believe that others will now expect them to do even better. Because attaining others' goals and standards will win respect and caring, the socially prescribed perfectionists have a fear of looking silly or stupid to others and have a strong need to be admired and respected. Because of the unrealistic nature of the perfection demanded, these individuals see a lot of failure in themselves and believe that others will see them as total failures and, as a result, that others will reject them. Following successes, the socially prescribed perfectionist believes that others will discount the success and focus on the flaws. Furthermore, they believe that it is even more important to perform even better now.
