# Supplementary material for: Other-Oriented Perfectionism in Children and Adolescents: Development and Validation of the Other-Oriented Perfectionism Subscale-Junior Form (OOPjr)
Source: J Psychoeduc Assess. 2022 Mar 5;40(3):327–45. doi: 10.1177/07342829211062009 (PMC9092920; doi:10.1177/07342829211062009)
Supplement: sj-pdf-2-jpa-10.1177_07342829211062009 – Supplemental Material for Other-Oriented Perfectionism in Children and Adolescents: Development and Validation of the Other-Oriented Perfectionism Subscale-Junior Form (OOPjr) [file sj-pdf-2-jpa-10.1177_07342829211062009.pdf]

**Table 2 (supplemental).**
*Factor loadings from the exploratory factor analysis for the initial item pool.*

| | Factor 1 | Factor 2 |
|---|---|---|
| *Other-oriented perfectionism* | | |
| 1. I do not like to be friends with anyone who is not perfect. | .98 | -.02 |
| 2. If other kids aren't perfect, I don't like them. | .79 | .18 |
| 3. People who want to be my friend need to be perfect. | .82 | .07 |
| 4. It is important that people I am close to are perfect. | .72 | .19 |
| 5. I need my family members to be perfect. | .84 | .01 |
| 6. Everything that others do must be perfect. | .48 | .46 |
| 7. I get upset when other kids aren't perfect. | .50 | .37 |
| 8. I need my friends to be perfect. | .69 | -.02 |
| 9. I think less of my classmates if they make mistakes. | .78 | -.06 |
| 10. I expect my friends to be the best, not second best. | .45 | .33 |
| 11. There is no excuse for the mistakes made by people around me. | .40 | .27 |
| 12. I don't like to be around people who don't try to be perfect. | .00 | .85 |
| 13. The people in my class should not make the mistakes they make. | .02 | .65 |
| 14. I expect other people to do their absolute best. | -.19 | .93 |
| 15. If I am trying to be perfect, others should also try to be perfect. | .03 | .82 |
| 16. The kids in my class should try harder to be perfect. | .05 | .82 |
| 17. I do not like it when other people do not try their best. | -.40 | .79 |

*Note*. All loadings are significant at $p < .001$.
