# Supplementary material for: Other-Oriented Perfectionism in Children and Adolescents: Development and Validation of the Other-Oriented Perfectionism Subscale-Junior Form (OOPjr)
Source: J Psychoeduc Assess. 2022 Mar 5;40(3):327–45. doi: 10.1177/07342829211062009 (PMC9092920; doi:10.1177/07342829211062009)
Supplement: sj-pdf-3-jpa-10.1177_07342829211062009 – Supplemental Material for Other-Oriented Perfectionism in Children and Adolescents: Development and Validation of the Other-Oriented Perfectionism Subscale-Junior Form (OOPjr) [file sj-pdf-3-jpa-10.1177_07342829211062009.pdf]

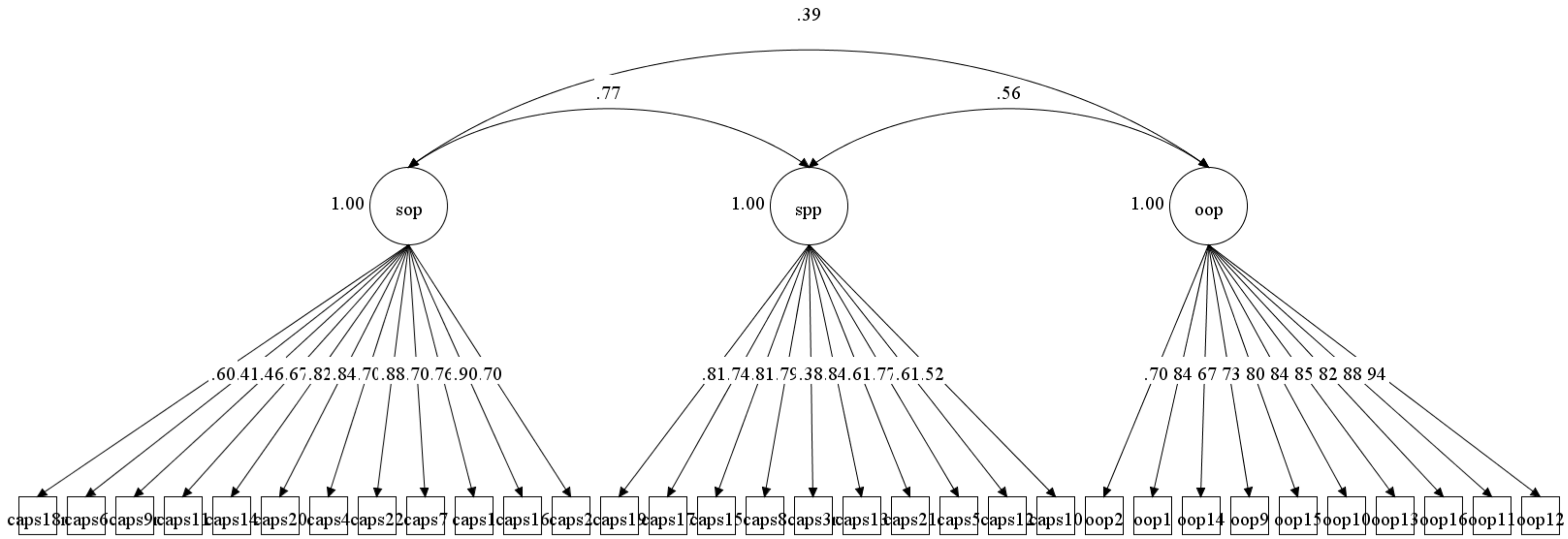

Figure 1 (supplemental). Measurement model. Ovals represent latent variables. Rectangles represent observed indicators. Estimates are standardized. Double-headed black arrows represent significant correlations (*p* < .05). Single-headed black arrows represent significant loadings (*p* < .05). **SOP** = self-oriented perfectionism; **SPP** = socially prescribed perfectionism; **OOP** = other-oriented perfectionism.
