# Supplementary material for: Other-Oriented Perfectionism in Children and Adolescents: Development and Validation of the Other-Oriented Perfectionism Subscale-Junior Form (OOPjr)
Source: J Psychoeduc Assess. 2022 Mar 5;40(3):327–45. doi: 10.1177/07342829211062009 (PMC9092920; doi:10.1177/07342829211062009)
Supplement: sj-pdf-4-jpa-10.1177_07342829211062009 – Supplemental Material for Other-Oriented Perfectionism in Children and Adolescents: Development and Validation of the Other-Oriented Perfectionism Subscale-Junior Form (OOPjr) [file sj-pdf-4-jpa-10.1177_07342829211062009.pdf]

Table 1 (supplemental).
Multi-trait multi-method matrix

		Parent ratings of child			Child self-report		
		OOP	SOP	SPP	OOP	SOP	SPP
Parent ratings of child	OOP	—					
	SOP	.40	—				
	SPP	.39	.61	—			
Child self-report	OOP	**.30**	.03	.12	(.86)		
	SOP	.16	**.39**	.28	.26	(.90)	
	SPP	.21	.24	**.25**	.44	.64	(.86)

Note. **OOP** = other-oriented perfectionism; **SOP** = self-oriented perfectionism; **SPP** = socially prescribed perfectionism. Reliabilities are in parentheses. Reliabilities for parent ratings of child not available due to parent ratings being single items. Heterotrait-monomethod correlations are in gray sub diagonals. Heterotrait-heteromethod correlations are enclosed by a broken line.
